# Impact of Polymer Chain Rearrangements in the PA Structure of RO Membranes on Water Permeability and *N*-Nitrosamine Rejection

**DOI:** 10.3390/molecules28166124

**Published:** 2023-08-18

**Authors:** Silvia Morović, Alegra Vezjak Fluksi, Sandra Babić, Krešimir Košutić

**Affiliations:** 1 Department of Physical Chemistry, Faculty of Chemical Engineering and Technology, University of Zagreb, Marulićev trg 20, 10000 Zagreb, Croatia; smorovic@fkit.unizg.hr (S.M.); avezjakfl@fkit.hr (A.V.F.); 2 Department of Analytical Chemistry, Faculty of Chemical Engineering and Technology, University of Zagreb, Marulićev trg 20, 10000 Zagreb, Croatia

**Keywords:** *N*-nitrosamines, polyamide RO membranes, *n*-propanol, pore size, pore size distribution

## Abstract

The use of solvents is overall recognized as an efficient method to improve the water permeability of polyamide thin film composite membranes (PA-TFC). The objective of this work was to test the performance of the membranes after exposing them to *n*-propanol (*n*-PrOH) to improve the permeability of the membranes while maintaining the rejection factor for small uncharged organic molecules, namely *N*-nitrosamines (NTRs). After the membranes were exposed to *n*-PrOH, the water permeability of the UTC73AC membrane increased by 98%, with minimal change in rejection. *N*-nitrosodiethylamine (NDEA) rejection decreased (3.4%), while *N*-nitrosodi-*n*-propylamine (NDPA) and *N*-nitrosodi-*n*-butylamine (NDBA) rejection increased by 0.9% and 2.8%, respectively. In contrast, for the BW30LE membrane, water permeability decreased (by 38.7%), while rejection factors increased by 14.5% for NDEA, 6.2% for NDPA, and 15.0% for NDBA. In addition, the morphology of the membrane surface before and after exposure to *n*-PrOH was analyzed. This result and the pore size distribution (PSD) curves obtained indicate that the rearrangement of polymer chains affects the network or aggregate pores in the PA layer, implying that a change in pore size or a change in pore size distribution could improve the permeability of water molecules, while the rejection factor for NTRs is not significantly affected.

## 1. Introduction

Many regions of the world are already facing water scarcity [1]. Rapid urbanization and increasing water demand require heavy investment in technologies that enable the reuse of municipal wastewater as a source of drinking water [2]. However, there are still many challenges associated with emerging contaminants and disinfection byproducts. In general, the formation of *N*-nitrosamines (NTRs) is a major concern in water treatment plants (WTPs) where chloramination is used, especially in potable reuse, where wastewater-impacted waters contribute to source waters [3,4]. NTRs, a class of aqueous nitrogen-containing disinfection byproducts, are classified by the U.S. Environmental Protection Agency (EPA) as probably carcinogenic to humans [5]. These polar substances are mostly soluble in water. Due to their hydrophilicity and low adsorption capacity, they can contaminate groundwater and consequently increase human exposure through their toxic and carcinogenic effects [6]. NTRs are most commonly formed during disinfection processes, i.e., during the reaction of oxidants (mostly chloramine, chlorine) with nitrogen-containing precursors [7,8]. *N*-nitrosodimethylamine (NDMA) is a frequently detected nitrosamine, usually formed at levels of ng L^−1^ during the chlorination and chloramination of water, although much larger amounts can also be formed in wastewater [9].

Currently, there are several strategies to treat and control NTRs. The first strategy focuses on reducing the amount of precursors of NTRs (prior to entering the WWTP or within the WWTP), thereby preventing the formation of these compounds through various treatment processes. The second strategy focuses on the optimization of the chloramination process, while the third strategy is based on the application of advanced processes to remove nitrosamines after their formation [10]. Considering that NTRs are often formed during the disinfection process, the improvement of treatment methods that allow removal of or reduction in NTR concentration is essential. There are many methods to remove NTRs, e.g., adsorption, enhanced coagulation, membrane processes of reverse osmosis, sunlight and UV photolysis, advanced oxidation process (AOPs), and various electrochemical and biological processes [11,12]. One of the most efficient technologies for NDMA removal is UV photolysis; however, its energy requirement makes the process uneconomical [13,14]. On the other hand, reverse osmosis (RO) is a widely used process for the production and treatment of drinking water and for wastewater treatment on an industrial scale due to its high separation efficiency. Modern water treatment systems, including membrane processes, consume little energy, so operating costs are relatively low, making them more economically viable than UV processes [15,16]. Fujioka et al. extensively investigated the retention of NTRs with RO membranes, and in one of their studies, they collected all available laboratory-scale data showing that the retention of NDMA by various RO membranes did not exceed 70% [17,18]. On the other hand, Fujioka et al. reported in a full-scale study that the retention of NDMA and *N*-nitrosodiethylamine (NDEA) was lower compared to the laboratory scale, ranging from 4 to 47% and 0 to 53%, respectively. They found that significant variations in the retention of NTRs compared to laboratory-scale studies are not only influenced by parameters such as different feed temperatures and fouling of the membrane, but also that flat membranes operated with very low water recovery are not necessarily comparable to full-scale operations [19]. Furthermore, to improve the retention factor of NTRs, they also investigated membrane plugging with linear-chain amines, amides, and epoxides along with membrane fouling, which was shown to have a positive effect on the retention factor [20]. However, increasing the resistance to the transfer of ions and molecules leads to a high retention factor for organic and inorganic molecules, but lowers water transport, i.e., lowers water permeability. This phenomenon is also known as the “trade-off” phenomenon [21,22]. To overcome this effect, great efforts have often been made to improve water permeability while maintaining or increasing the retention factor. One of the ways to improve membrane permeability is post-treatment with various organic solvents such as short-chain monohydric alcohols, benzyl alcohol, dimethylformamide (DMF), etc. [23,24,25].

The mechanism of mass transfer, the interactions of solvents with polymeric materials, and their effects on the structure of polymeric materials are still not fully understood and represent a major challenge for progress in the fabrication of new membrane materials. The contact between organic solvents and the polyamide (PA) active layer of thin film composite (TFC) RO membranes depends largely on the interactions of the solvent with the PA. One of the parameters used to evaluate the interactions between polymers and solvents is the Hildebrand parameter, which describes the ability of solvents to act as swelling agents [26]. However, according to Shin et al., the Hildebrand solubility parameter does not predict the swelling of polar materials as well as nonpolar materials, so they propose the Hansen solubility parameter as a parameter for evaluating the solvent activation of RO membranes [25]. The Hansen parameter (*R*_a_) is also a parameter used to predict the solubility of polymers in solvents, but unlike the Hildebrand parameter, it takes into account the contribution of intermolecular interaction, i.e., dispersion force (*δ*_d_), polar force (*δ*_p_), and hydrogen bonding force (*δ*_h_), and is calculated according to the following equation: *R*_a_^2^ = 4(*δ*_d2_ − *δ*_d1_)^2^ + (*δ*_p2_ − *δ*_p1_)^2^ + (*δ*_h2_ − *δ*_h1_)^2^, where a lower *R*_a_ value indicates better solubility [27]. 

Low-molecular-weight polymer fragments in membranes may dissolve upon contact with solvents, or swelling of PA may occur, depending on the strength of the solvent. In some cases, it has been shown in the literature that the contact of certain solvents with membranes improves the water permeability of RO membranes, while the rejection factor does not change or changes only slightly. However, in some cases, the free volume between the polymer chains can increase greatly, allowing the chain segments to move and rotate more freely, leading to plasticization, which can result in a loss of PA structure and changes in separation properties [28]. The study by Shin et al. showed that the use of mild solvents such as ethanol (*R*_a_ = 12.7 MPa^1/2^) and isopropanol (*R*_a_ = 11.2 MPa^1/2^), as well as DMF (*R*_a_ = 4.0 MPa^1/2^), which has higher solvency power (indicator of the swelling ability), can increase the water permeability but causes the irreversible deformation of the PA layer, resulting in lower NaCl rejection. However, using a solvent of appropriate solvency power, such as benzyl alcohol (*R*_a_ = 8.1 MPa^1/2^), swelling of the PA active layer can be achieved with minimal structural deformation, resulting in higher permeability (about 140%) of the RO membranes without significant effects on the NaCl retention factor [25]. On the other hand, Mukherjee et al. showed that short-term treatment with isopropyl alcohol can simultaneously improve permeability and NaCl retention factor, explaining that the process leads to polymer chain dissolution coupled with surface tension driven collapse of pores [29]. Furthermore, Gorgojo et al. showed that after the interfacial polymerization process, post-treatment with DMF can increase the retention factor for membranes whose initial retention was below 90%. In their work, they concluded that the swelling of the PA layer can cause healing of non-selective pathways that redistribute the polymer matrices. Moreover, it is possible that blocking defects with dissolved PA fragments of medium and large molecular weight is responsible for such an effect, which increases the retention factor of NaCl. The increase in water permeance, from 0.2 to 1.6 L m^−2^ h^−1^ bar^−1^, probably occurs due to the dissolution of the loose PA layer, reducing the thickness of the membrane or clearing the pathways through which water can pass [30]. Shi et al. showed that solvent activation on the newly prepared RO membrane largely depends on the solvency power of solvent by comparing the effect of the polar solvent ethanol and the nonpolar solvent hexane during post-treatment. The use of ethanol resulted in higher water permeability, but a slightly lower retention factor of NaCl was obtained compared to hexane. They also compared different nonpolar solvents and concluded that membrane performance is also affected by the viscosity of the solvent, i.e., the length of the carbon chain. Therefore, the swelling capacity is greater with shorter carbon chains. Moreover, it was shown that activation time plays an important role when nonpolar solvents are used, since more nonpolar molecules remain in the PA network during longer contact and hinder the transport of water and salt [31].

In summary, depending on the solvent power, polymer chain disruption and size reduction of the primary nanoscale building blocks in the PA layer can occur, leading to a change in pore size distribution. Guided by these conclusions, in our previous preliminary research, we tested five commercially available RO membranes and exposed them to alcohols with different Hansen solubility parameters at different time intervals, since the exposure time can affect the transport of water and salt molecules due to the residual solvent molecules in the PA layer. Furthermore, in our preliminary studies, exposure of PA membranes to *n*-PrOH had no significant effect on the rejection factor, but a significant effect on the water permeability of some membranes was observed [32]. Therefore, this study investigated the effects of exposure to *n*-PrOH on three commercially available RO membranes (BW30LE, ACM1, UTC-73AC) along with the effects of *n*-PrOH on the structure of the PA layer. The performance of each membrane was evaluated before and after short-term exposure to alcohol. The rejection factor was determined using NaCl and CaCl_2_ salts and *N*-nitrosamines. Since NTRs are small molecules that are not ionized in the pH range of 6 to 8, the rejection of NTRs is mainly governed by the size exclusion mechanism, i.e., rejection depends on the pore size in the PA layer and the molecular size of the solutes, making them suitable for studying the structure of PA.

## 2. Results

### 2.1. Improvement of Water Permeability and N-Nitrosamine Rejection

**Figure 1:**
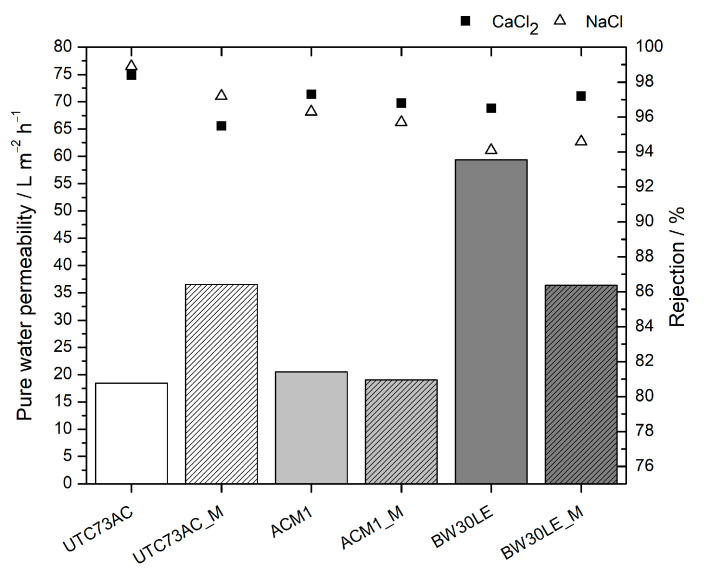
Pure water permeability and NaCl, CaCl_2_ rejection factor of pristine and *n*-PrOH-treated membranes (“M” stands for membranes after exposure to *n*-PrOH).

Water permeability and rejection of salts for selected RO membranes are presented in Figure 1. It can be seen that pristine RO membranes UTC73AC and ACM1 have similar water permeabilities, while the BW30LE membrane has an almost three times higher water permeability, which may be a consequence of the different structure of the PA layer. Among the selected RO membranes, ACM1 showed the highest rejection of NTRs, 93.8%, 97.4%, and 95.6% for NDEA, *N*-nitrosodi-*n*-propylamine (NDPA), and *N*-nitrosodi-*n*-butylamine (NDBA), respectively, but lower water permeability (20.52 L m^−2^ h^−1^), and BW30LE showed the lowest rejection of 75.7%, 89.6%, and 86.1% for NDEA, NDPA, and NDBA, respectively, but the highest water permeability (59.38 L m^−2^ h^−1^). 

**Figure 2:**
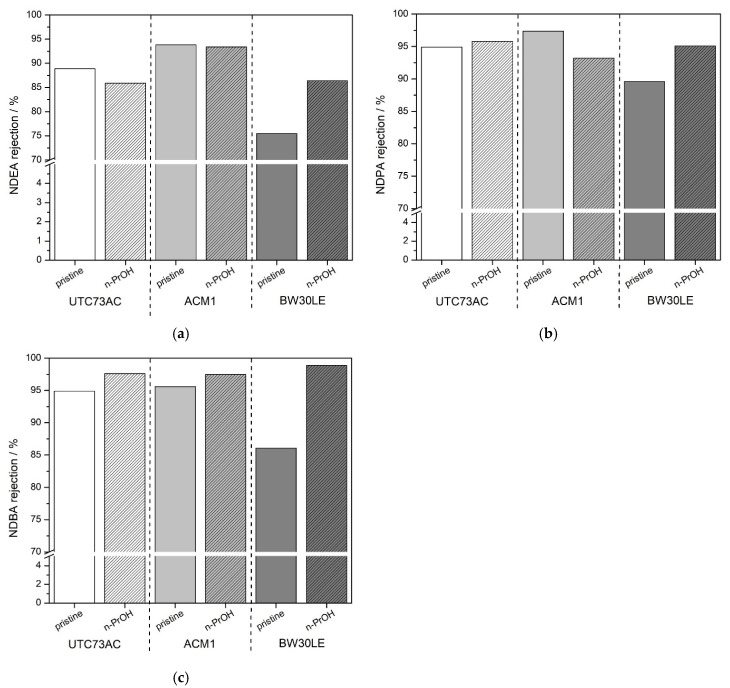
Rejection of NDEA (**a**), NDPA (**b**), NDBA (**c**) by pristine and *n*-PrOH-treated membranes.

After the membranes were exposed to *n*-PrOH, the water permeability of UTC73AC membrane increased 98% with minimal change in rejection. The rejection of NDEA decreased slightly from 88.9 to 85.9%, while the rejection of NDPA and NDBA increased by 0.9% and 2.8%, respectively (Figure 2). For the ACM1 membrane, water permeability decreased slightly (7%), while the rejection of NDEA did not change significantly and the rejection of NDBA improved slightly (2%) (Figure 2). In contrast, for the BW30LE membrane, water permeability decreased (by 38.7%), while NTRs rejection improved significantly. The rejection factor for all nitrosamines increased from 75.5 to 86.4% for NDEA, from 89.6 to 95.1% for NDPA, and from 86.1 to 98.9% for NDBA (Figure 2). Enhanced performance of UTC73AC in terms of permeability and of BW30LE in terms of NTRs rejection factor can be attributed to the morphological change in the polyamide separation layer after exposure to *n*-PrOH.

In contrast to NTRs, conductivity rejection for selected membranes showed a similar trend after exposure to *n*-PrOH, but the difference was much smaller. For the ACM1 membrane, conductivity rejection decreased by <0.6%, whereas for the UTC73AC membrane, it decreased by 1.7% and 3.0% for NaCl and CaCl_2_, respectively. For the BW30LE membrane, rejection increased by <0.7%. Such a result is expected since the permeation of charged particles is not only governed by size exclusion but also by electrical repulsion [33]. From Figure 1, it can be concluded that the BW30LE membrane is slightly more electrically charged than the UTC73AC and ACM1 membranes since ∆*R* is higher for NaCl and CaCl_2_ solutions than for the other membranes. According to the literature, the changes in conductivity rejection depend on changes in the structure of the PA layer and the charge on the membrane surface [18]. The results obtained, which differ from those for NTR, suggest that changes in the charge on the membrane surface may have occurred, but no definitive conclusions can be drawn, as the zeta potential of the membranes was not measured in this work.

### 2.2. ATR-IR

**Figure 3:**
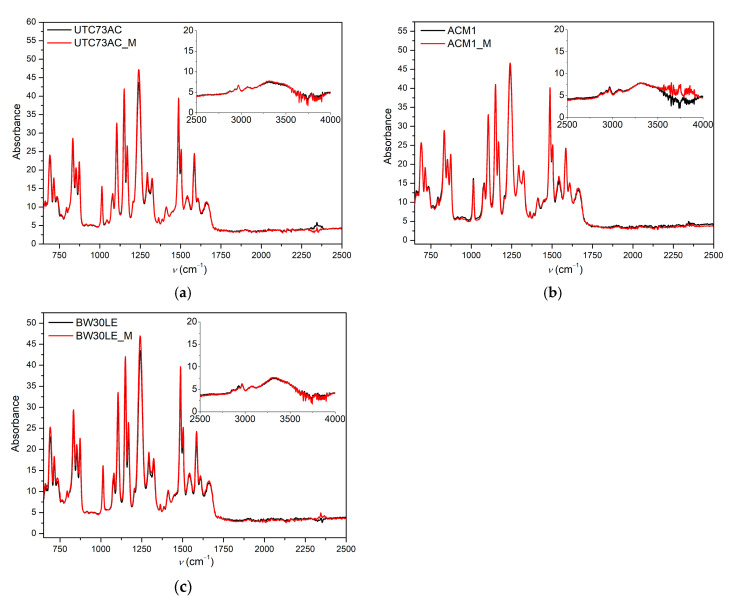
Infrared spectra of pristine and *n*-PrOH-treated UTC73AC (**a**), ACM1 (**b**), and BW30LE (**c**) (“M” stands for membranes after exposure to *n*-PrOH).

**Figure 4:**
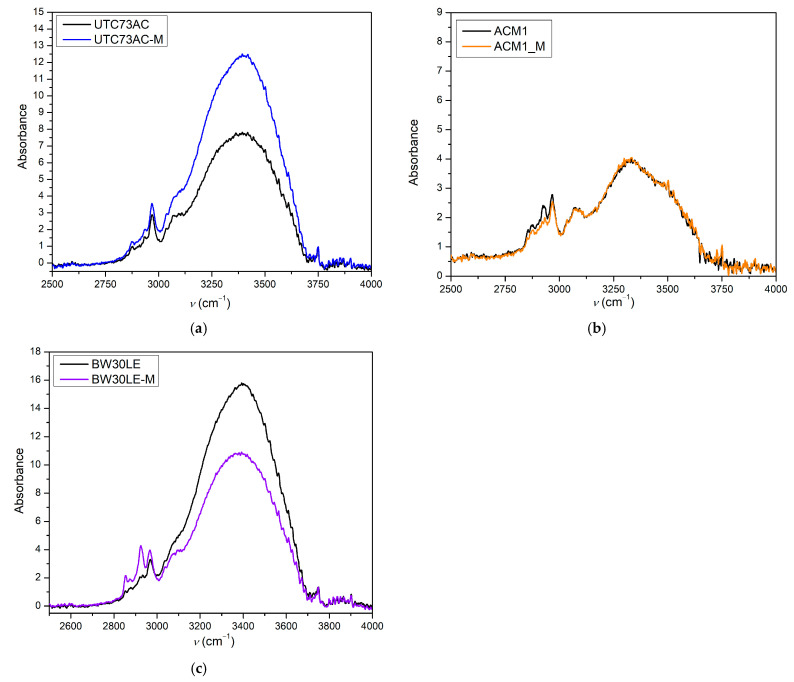
Infrared spectra of pristine and *n*-PrOH-treated UTC73AC (**a**), ACM1 (**b**), and BW30LE (**c**) membranes after swelling in water (“M” stands for membranes after exposure to *n*-PrOH).

Infrared spectroscopy was used to analyze the chemical composition and water content of selected RO membranes before and after exposure to *n*-PrOH. The analyzed infrared spectra are presented in Figure 3 and Figure 4.

Typical bands for polysulfone are 1365 cm^−1^ (C-H deformation vibrations of >C(CH_3_)), 1280–1350 cm^−1^, and 1180–1145 cm^−1^ (corresponding to the asymmetric and symmetric stretching vibrations of SO_2_ group, respectively). Moreover, the 1487, 1504, and 1587 cm^−1^ bands correspond to the in-plane stretching vibrations of the aromatic ring. As for the recognizable polyamide bands, the characteristic band at 1660 cm^−1^ is attributed to the C=O stretching as the dominant contribution in the amide I band, the C-N stretching and the C-C-N deformation vibration in a secondary amide group. The band at 1609 cm^−1^ is attributed to the N-H deformation vibration or the C=C stretching vibration in an aromatic amide. The in-plane N-H bending vibration and the N-C stretching vibration of a -CO-NH group is located at 1541 cm^−1^ [34]. As can be seen in Figure 3, all the bands mentioned are present in the infrared spectra of pristine membranes and those exposed to *n*-PrOH. From the FTIR spectra presented, it is evident that no change in the intensity of the peaks characteristic of PA can be seen for the UTC73AC, ACM1, and BW30LE membranes, since *n*-PrOH is not expected to dissolve the PA layer.

In addition, Figure 4 shows the ATR-IR spectra of pristine and *n*-PrOH-treated membranes that were soaked in DI for 24 h before analysis. The intensity of the signal from the OH groups (3400 cm^−1^) was analyzed to determine the effects of exposure to *n*-PrOH on the water content of the membranes. For the UTC73AC membrane, it can be seen that the intensity of the broad peak increased 1.5-fold, with a maximum at 3400 cm^−1^ (OH stretching), whereas for BW30LE, it decreased after contact with *n*-PrOH and was 0.7 of the original intensity. There was no change in peak intensity for the ACM1 membrane. According to Kolev and Freger, water uptake in the PA layer depends on two phenomena: the filling of the permanent voids with water and the osmotic swelling of the nonporous fraction. They found that the thickness of the PA layers increases by 10–11% after swelling, which means that the density of the PA layer decreases after hydration; thus, the presence of voids affects the density and water uptake [35]. Since, in our study, the PA layer was not separated from the polysulfone and the support, the increase in peak intensity cannot be solely attributed to a change in the structure of the PA layer. Therefore, the change in peak intensity indicates that the water uptake of the UTC73AC membrane increased after contact with *n*-PrOH, while the water uptake of BW30LE is slightly lower. This observation is consistent with the water permeability values obtained before and after contact with *n*-PrOH, which directly indicates a structural change within the membrane.

### 2.3. Contact Angle

**Figure 5:**
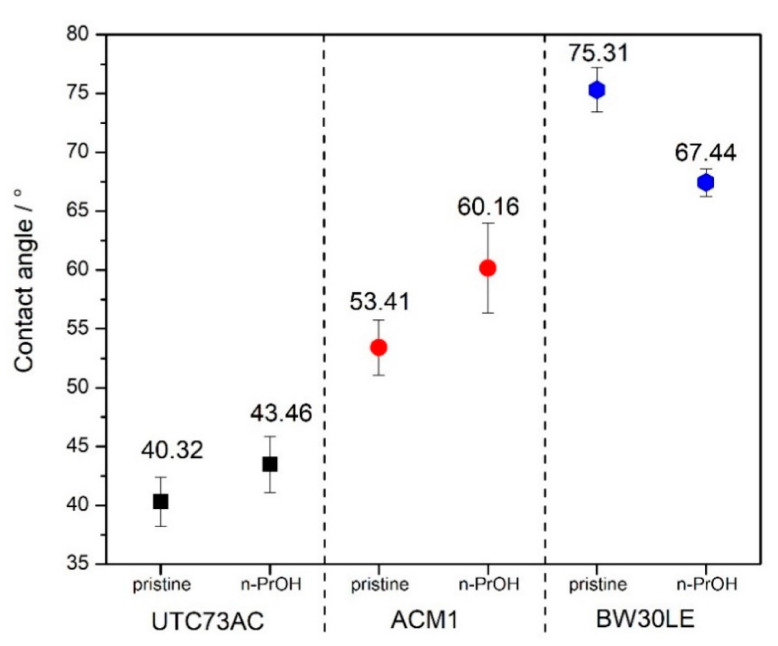
Water contact angle for pristine and *n*-PrOH-treated membranes.

The change in the surface hydrophilicity/hydrophobicity of the selected RO membranes after exposure to *n*-PrOH was analyzed via contact angle measurement with deionized water. Figure 5 shows how the exposure to *n*-PrOH affected the contact angle of the tested membranes. For UTC-73AC and ACM1, the contact angle changed slightly from 40.32° to 43.45° and from 53.41° to 60.16°, respectively, while for the BW30LE membrane, a decrease in the contact angle from 75.31° to 67.44° was observed. These observations could be correlated with the decrease in hydrophilic and negatively charged carboxyl groups on the surface of the PA layer [30]. The contact with solvents can lead to a deformation of the structure of the PA layer, i.e., it can cause a decrease in the density of the carbonyl groups, leading to an increase in the hydrophobicity of the PA layer.

### 2.4. Pore Size Distribution

**Figure 6:**
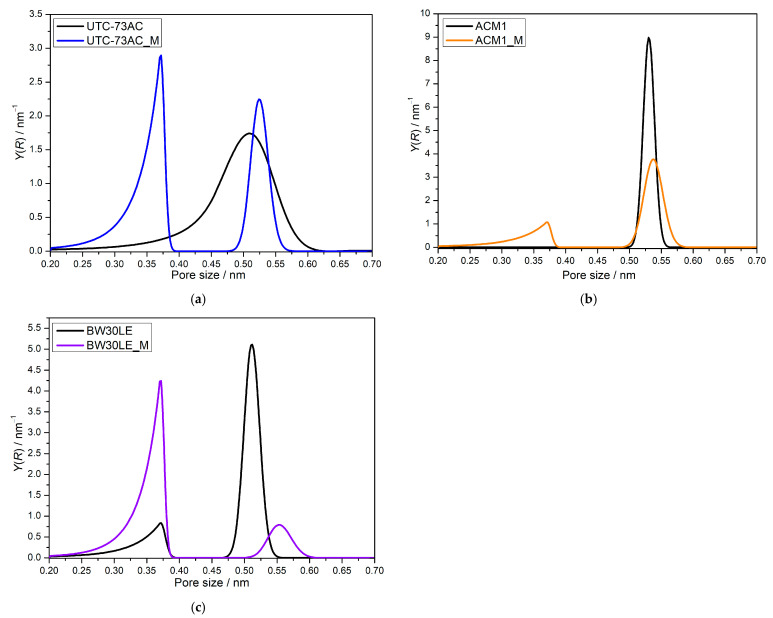
Pore size distribution of pristine and *n*-PrOH-treated UTC73AC (**a**), ACM1 (**b**), and BW30LE (**c**) (“M” stands for membranes after exposure to *n*-PrOH).

The porosity of UTC73AC, ACM1, and BW30LE was investigated before and after exposure to *n*-PrOH, and the pore size distributions (PSD) obtained are presented in Figure 6.

It can be seen that the PSDs obtained are different for all the membranes used. The BW30LE membrane shows a bimodal distribution, with the first maximum in the range of 0.25–0.40 nm having a broader distribution and lower intensity than the second peak, which is in the range of 0.47–0.57 nm (Figure 6c). ACM1 shows a unimodal distribution, and the maximum of the curve is in the range of 0.49–0.56 nm (Figure 6b). In contrast, the PSD curve of the UTC-73AC membrane differs from the others and shows a much broader distribution in the range of 0.20–0.63 nm (Figure 6a). After exposing the membrane to *n*-PrOH, it is obvious that the pore size distribution changed. For BW30LE, it can be seen that the intensity of the first peak increases in the range of 0.25–0.40 nm, while the intensity of the second peak decreases significantly and shifts slightly to larger values (0.50–0.61 nm). Furthermore, the ACM1 membrane showed a bimodal distribution, where the intensity of the maxima decreased in the range of 0.49–0.59 nm and a small intensity peak appeared in the range of 0.20–0.40 nm. Moreover, a bimodal distribution with two maxima with higher intensity than the original broad peak was formed after exposure of UTC73AC to *n*-PrOH. The first peak with higher intensity is located at 0.20–0.40 nm, while the second peak with lower intensity is located at 0.47–0.58 nm. To compare the rejection of small neutral molecules, the molecular radius of NDEA, NDPA, and NDBA was compared with the changes in pore size distribution (Table 1). According to the literature, NDEA, NDPA, and NDBA have radii of 0.278, 0.295, and 0.302 nm, respectively [36].

All the selected membranes showed relatively good rejection of NTRs. The best removal efficiency for all NTRs was obtained with the ACM1 membrane due to its unimodal distribution with a maximum peak at 0.53 nm. Bimodal BW30LE showed slightly lower rejection, which might be related to a slightly broader distribution in the range of 0.47–0.57 nm. After exposure to *n*-PrOH, the rejection of the NTRs changed. In the case of BW30LE, the rejection factors increased for all NTRs. For UTC73AC, the rejection factor decreased for NDEA (3.4%), while it increased by 0.9% for NDPA and by 2.8% for NDBA. For ACM1, the rejection of NDEA did not change significantly, whereas the rejection of NDBA improved slightly (2%). The results obtained can be correlated with the changes in pore size distribution and will be discussed in the next section. 

### 2.5. AFM

**Figure 7:**
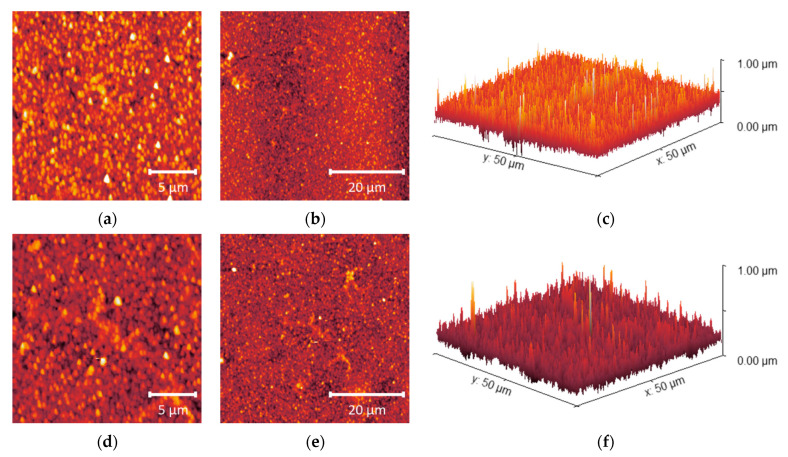
AFM microphotographs of pristine UTC73AC (**a**–**c**) and *n*-PrOH-treated UTC73AC (**d**–**f**); from left to right—20 × 20; 50 × 50 μm; 3D view of 50 × 50 μm microphotograph.

**Figure 8:**
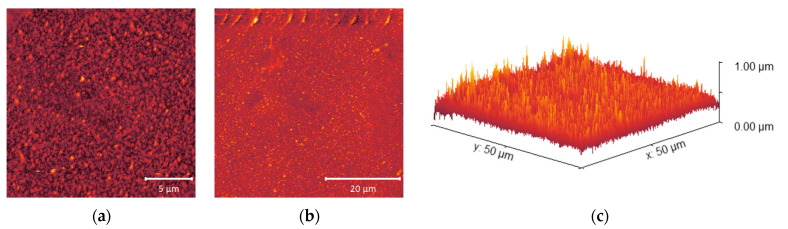

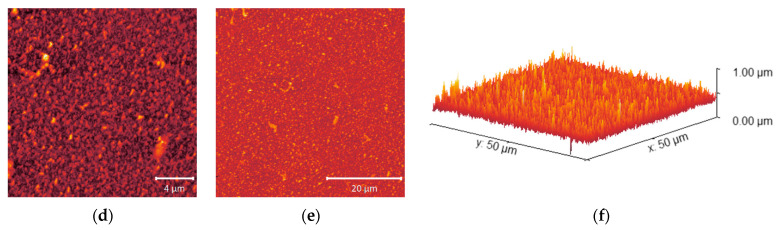
AFM microphotographs of pristine ACM1 (**a**–**c**) and *n*-PrOH-treated ACM1 (**d**–**f**); from left to right—20 × 20; 50 × 50 μm; 3D view of 50 × 50 μm microphotograph.

**Figure 9:**
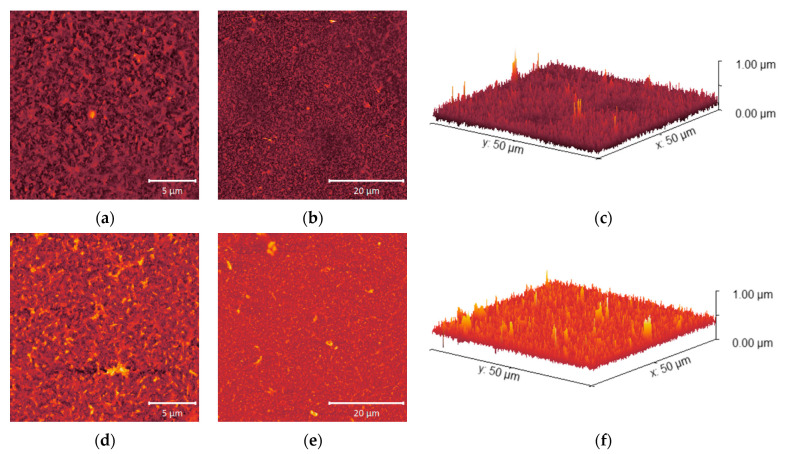
AFM microphotographs of pristine BW30LE (**a**–**c**) and *n*-PrOH-treated BW30LE (**d**–**f**); from left to right—20 × 20; 50 × 50 μm; 3D view of 50 × 50 μm microphotograph.

Figure 7, Figure 8 and Figure 9 show the surface roughness of the three RO polyamide membranes based on AFM measurements.

Among the obtained values, ACM1 has the highest surface roughness of 53.3 nm, the roughness of the BW30LE membrane was 52.8 nm, while the UTC73AC membrane (37.3 nm) had the lowest surface roughness compared to the other membranes (Table 2). In the 3D microphotographs of the studied membranes, an increase in roughness after exposure to *n*-PrOH can be seen for all selected membranes. In the case of the pristine UTC73AC membrane, a homogeneous distribution of bright spots representing the elevations on the membranes can be seen, while a larger number of dark patterns representing valleys on the membrane can be observed after exposure to *n*-PrOH (Figure 7). The valleys can be considered as internodular regions of the dense membranes. The surface roughness of UTC73AC changed significantly and reached a value of 48.0 nm. A similar topology was observed for ACM1 and BW30LE (Figure 8 and Figure 9), which had a roughness of 86.8 nm and 58.4 nm, respectively, after exposure to *n*-PrOH (Table 2).

### 2.6. SEM

**Figure 10:**
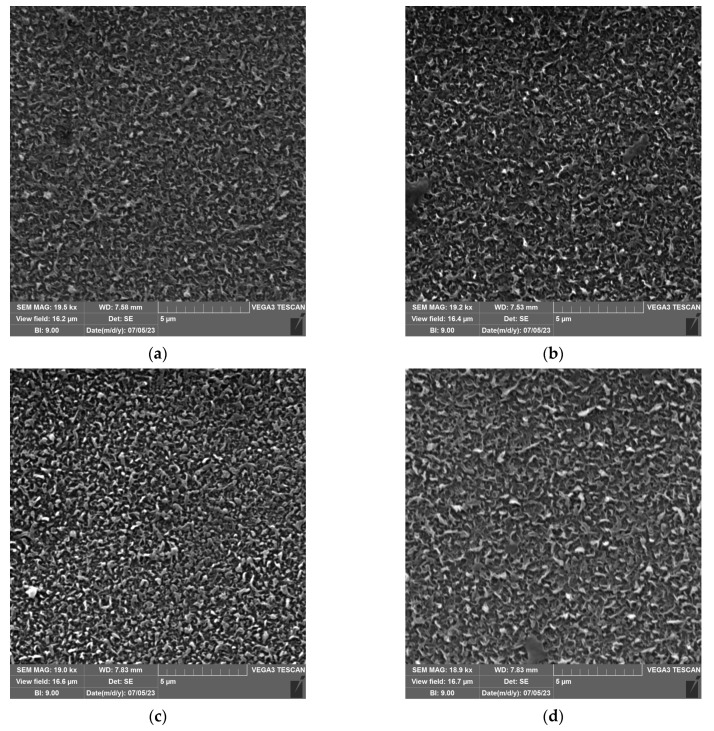

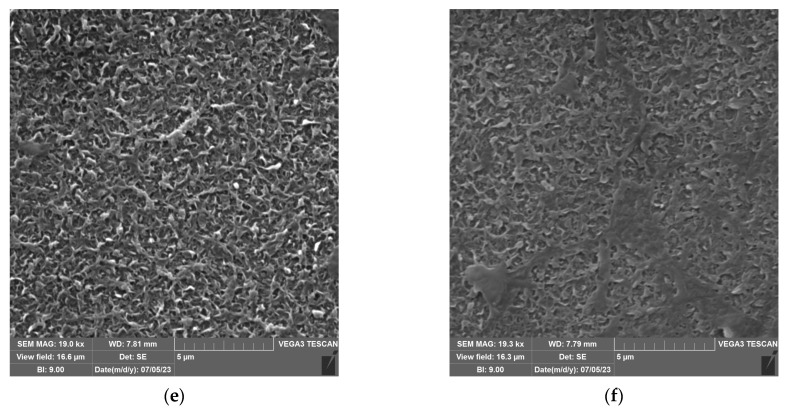
SEM images of pristine UTC73AC (**a**), ACM1 (**c**), and BW30LE (**e**) and *n*-PrOH-treated UTC73AC (**b**), ACM1 (**d**), and BW30LE (**f**) membrane surfaces.

The changes in the surface morphology of the pristine and *n*-PrOH-treated membranes are shown in Figure 10. In the micrographs of the pristine membranes, it is clear that the surface of the UTC73AC (Figure 10a) and BW30LE (Figure 10e) structure consists of more branched polymer strands, a “ridge-and-valley” morphology, whereas the ACM1 membrane (Figure 10c) has a packed, sphere-like nodular structure. The surface of the BW30LE membrane looks “looser” compared to the ACM1 and UTC73AC membranes, which appear denser and is consistent with the fact that the BW30LE membrane showed the highest water permeability.

After exposure to *n*-PrOH, the nanostructure of the original membranes changed significantly in the case of ACM1 (Figure 10d) and BW30LE (Figure 10f). It can be observed that the ACM1 membrane changed from the initial dense sphere-like nodular structure to a structure with more irregular polymer strands, a leaf-like morphology. In addition, BW30LE appeared to form a denser structure of polymer strands after exposure to *n*-PrOH. However, in the case of the UTC73AC (Figure 10b) membrane, no significant difference in structure can be seen in the micrographs.

## 3. Discussion

The changes in the polyamide structure depend on the thermodynamic quality of the solvent, i.e., the interactions of the solvent with the PA. In this study, *n*-PrOH was selected based on our preliminary studies and the Hansen solubility parameter. The Hansen solubility parameter of *n*-PrOH (11.5 MPa^1/2^) indicated that only physical changes can be expected in membranes after contact with *n*-PrOH, such as a physical rearrangement, i.e., a change in polymer chain entanglement, which consequently need not affect solute rejection but may increase the permeation of water molecules. In our study, we obtained an increase in water permeability in the case of UTC73AC without significant deterioration of the rejection factor, while the rejection factor was improved in the case of BW30LE and was accompanied by a decrease in water permeability. In order to better interpret the results of the selected membranes, the pore size distribution was calculated using the theoretical Surface Force–Pore Flow (SF-PF) model for the pristine membranes and for the membranes after contact with *n*-PrOH. A recent study by Wang et al. confirmed that water transport is driven by a pressure gradient within membranes, in contrast to the classical solution–diffusion model, where the main driving force is the water concentration gradient. In their experiments and simulations, they showed that water molecules are transported in chains through interconnected channels within the RO membranes, suggesting a pore flow mechanism rather than solution diffusion [37]. In addition, Dražević [38] and Freger [39] have shown that the rejection of organic molecules and conductivity rejection depend on the membrane properties (free volume, hole size, surface charge). Therefore, the surface morphology was analyzed to explain the given changes in performance.

The UTC73AC membrane showed a 98.0% increase in water permeability, while ACM1 and BW30LE decreased by 7.2 and 38.7%, respectively. Such variations could have arisen for several reasons: changes in pore size or pore size distribution and changes in PA layer thickness [40,41] From the obtained PSD curves, it can be observed that the largest change occurred in the pore distribution of the UTC-73AC membrane, as two peaks in the range of 0.20–0.40 nm and 0.47–0.58 nm emerged from a peak with low intensity but broad distribution (Figure 6a). The former could indicate a greater formation of smaller pores, in contrast to the pore distribution of the pristine membranes. However, it should be noted that the latter peak was slightly shifted towards larger pore sizes but had a narrower distribution, which could be correlated with the achieved water permeability and a slight decrease in NDEA, NaCl, and CaCl_2_ rejection factor (3.4%, 1.7%, and 2.9%, respectively). From the intensities obtained, the distribution of pores, and the significant increase in water permeability, it can be concluded that a more porous structure of the PA layer was formed, consisting of smaller pores. In Figure 6a, it can be seen that the second peak that occurred has a slightly higher intensity and a narrower distribution than the peak at the pristine membrane, so the separation of larger *N*-nitrosamines can be associated with the aforementioned phenomenon. Since more pores with a maximum at 0.52 nm were formed after activation, it is possible that a larger number of NDEA molecules with a radius of 0.278 nm pass through the newly formed pores, resulting in a smaller decrease in the rejection factor. For NDPA and NDBA, whose radii are larger, an increase in the rejection factor can be observed. The observed effect is consistent with the observation of Košutić and Kunst, who found that the rejection of small non-ionized organic pollutant molecules by the tight-pore membranes is influenced by both solute parameters and pore size; therefore, it cannot be said that either mechanism predominates [42]. For the ACM1 membrane, the appearance of a new peak was observed in the range 0.20–0.40 nm, accompanied by a decrease in peak intensity in the range 0.49–0.59 nm and a minimal shift toward larger pore sizes, which can be associated with a small decrease in water permeation (7%) and a slight decrease in the rejection factor for NDEA (0.5%) (Figure 6b). Despite the formation of smaller pores in the ACM1 membrane, there was a slightly broader distribution of pores in the range of 0.49–0.59 nm. For this reason, the rejection factor for small molecules, such as NDEA, may decrease by 0.5%. Also, the decrease in the intensity of the peak in this range could be the reason for the small decrease in the water permeability by 7%. Another effect was observed in the case of the BW30LE membrane, where it is possible that the polymer chains rearranged and formed a denser structure, which simultaneously reduced water permeability. Upon contact of the BW30LE membrane with *n*-PrOH (Figure 6c), an increase in the number of smaller pores was observed in the range of 0.25–0.40 nm, while a decrease in the number of pores was observed in the range of 0.50–0.61 nm. Due to the formation of a larger number of smaller pores, the change in pore size distribution was accompanied by an increase in the rejection factor for NDEA (14.5%), NDPA (6.2%), NDBA (15.0%), NaCl (0.5%), and CaCl_2_ (0.7%) and a decrease in permeability (38.7%). Since there was a significant decrease in water permeability, and a significant increase in the rejection factor after the exposure of BW30LE membrane to *n*-PrOH, it can be concluded that the reason for this is the occurrence of a new pore distribution, i.e., the occurrence of a large number of pores in the area 0.25–0.40 nm, while the remaining part of the pores in the area 0.50–0.61 nm is very small, which probably leads to the above-mentioned effect. Such observations are in good agreement with previous findings by Košutić et al., who showed on several membranes that a change in the effective number of pores affected the water permeation rate [43]. 

The surface roughness of the membranes increased after exposure to *n*-PrOH, with a rougher surface observed in all exposed membranes. This is confirmed by the root mean square *S*_q_, which was calculated in the scan area of 50 µm × 50 µm and is shown in Table 2. Changes in surface roughness are often correlated with changes in water permeability, whereas greater roughness indicates a greater surface area [44,45]. In contrast, in our study, higher surface roughness was observed in all cases after exposure of the membranes to *n*-PrOH, whereas water permeability increased only for UTC73AC. Such results are consistent with previous literature findings [45,46]. Freger emphasized the importance of the structure of the PA layer over surface roughness. According to Freger, the PA layer actually consists of two oppositely charged layers, with the negative charge predominating in the carboxyl-rich part and the opposite charge in the carboxyl-free part. The upper layer is looser and more porous, while the lower is much denser and separated by a sharp boundary deep inside the PA layer. In his study comparing different PA layers of high-pressure and high-flux RO membranes, he showed that the thickness of the carboxyl-free part of the PA layer, located deep inside the skin, mainly determines the water permeability, i.e., a higher porosity of the PA layer and a thinner dense barrier are responsible for the excellent permeability of high-flux membranes [45]. Furthermore, SEM images of the upper surface showed the changes in the morphology of the *n*-PrOH-exposed membranes. As can be seen from the micrographs (Figure 10), the original structure of UTC73AC, ACM1, and BW30LE was changed. Song et al. showed that the performance of the membrane strongly depends on the roughness features. They showed how the change from balloon-like nodules on the membrane surface to leaf-like and donut-like roughness features can greatly affect the water permeability [47]. When we summarize the SEM and AFM analysis, it is obvious that the rearrangement of PA layer occurred in all cases, which contributed to the change in membrane performance. Moreover, surface roughness is often associated with an increase in contact angle [48]. However, in our study, an increase in contact angle was observed for the ACM1 and UTC73AC membrane, while the contact angle decreased for the BW30LE membrane. Contact angle measurements can provide information on various surface properties such as hydrophilicity, surface wettability, surface charge, interaction energy, etc. Van der Bruggen has shown that due to the clustering effect, hydrophilic membranes can become more hydrophobic and vice versa [49]. This effect can be seen in Figure 5 and can be caused by a rearrangement of the polymer structure. Additionally, swelling can affect the rearrangement of the polymer chains and therefore affect the network or aggregate pores in the PA layer. According to Dražević et al., water permeability can be correlated with intrinsic swelling. RO membranes that have more “fluffy” parts in the PA layer can expand isotropically during swelling in water, i.e., expand in all three dimensions. In contrast, dense PA films can expand in only one dimension, i.e., the dimension of thickness [38]. Considering the above, swelling can affect the formation of a connected network of pores in different ways. This hypothesis was verified using IR, which showed that water uptake changed after exposure to *n*-PrOH, whereas the change in peak intensity at 3400 cm^−1^ indicated that the water uptake of the UTC73AC membrane increased after contact with *n*-PrOH, while the water uptake of BW30LE decreased (Figure 4). 

In view of the above, it can be concluded that the treatment of membranes with suitable solvents could significantly improve the performance of existing RO membranes, which would be beneficial for their wider industrial application in *N*-nitrosamine separation.

## 4. Materials and Methods

### 4.1. Materials

The chemicals used were of analytical grade: *n*-propanol (≥99%, VWR Chemicals BDH Prolabo, Rosny-sous-Bois, France), sodium chloride (p.a., Lach-Ner, Neratovice, Czech Republic), calcium chloride (p.a., Lach-Ner, Neratovice, Czech Republic). *N*-nitrosamines used were as follows: *N*-nitrosodiethylamine (99%, Aldrich, St. Louis, MO, USA), *N*-nitrosodi-*n*-propylamine (99.9%, Supelco, St. Louis, MO, USA) and *N*-nitrosodi-*n*-butylamine (99.9%, Supelco, St. Louis, MO, USA). HPLC-grade acetonitrile (J. T. Baker, Deventer, The Netherlands) and ultra-pure water prepared using the Milli-Q^®^ Reagent Grade Water System (Millipore Corporation, Bedford, MA, USA) were used for chromatographic analysis. Organic compounds selected as markers were as follows: trimethylene oxide (97%, Acros Organics, Morris Plains, NJ, USA), 1,3-dioxolane (99.8%, Sigma-Aldrich, St. Louis, MO, USA), 1,4-dioxane (99.8%, Sigma-Aldrich, St. Louis, MO, USA), 12-crown-4 (98%, Fluka, Buchs, Switzerland), 15-crown-5 (98%, Merck-Schuchardt, Hohenbrunn, Germany), and 18-crown-6 (99.5%, Fluka, Buchs, Switzerland). Commercial RO membranes BW30LE (DOW FILMTEC™, Wilmington, DE, USA), UTC-73AC (Toray™, Poway, CA, USA) and ACM1 (TriSep™, Goleta, CA, USAwere supplied from Sterlitech Corporation, Auburn, WA, USA as flat sheets and stored dry before use. Nominal membrane characteristics provided by the manufacturer are given in Table 3.

### 4.2. n-PrOH Treatment of Membranes

To evaluate the effects of *n*-PrOH on membrane performance, membranes were first rinsed with distilled water (DI) and left in DI water for 24 h. Afterwards, the membranes were immersed in *n*-PrOH and left for 60 min. After contact with *n*-PrOH, the membranes were washed thoroughly with DI and stored in DI for 16 h before use.

### 4.3. Characterization of PA-TFC Membranes

#### 4.3.1. RO Performance

**Figure 11:**
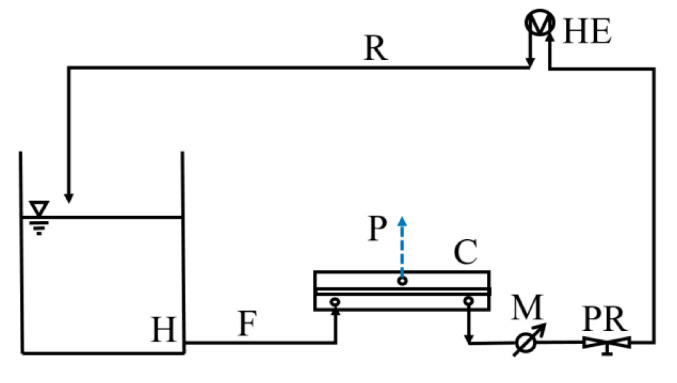
Schematic representation of the RO set up Sterlitech SEPA II cell. H: hold up tank, M: manometer, HE: heat exchanger, PR: pressure regulator, C: membrane cell. F, P, and R stand for feed, permeate, and retentate, respectively.

Experiments were performed in a Sepa CF II cell cross-flow apparatus (Sterlitech Corporation, USA) with a membrane area of 1.38 × 10^−2^ m^2^, channel dimensions of 14.5 × 9.5 × 0.17 cm^3^ (length × width × height), and a spacer of 0.45 mm (Figure 11). Solutions were pumped through the system using the Hydracell DO3SASGSSSCA pump (Wanner Engineering Inc., Minneapolis, MN, USA) and circulated at a rate of 1.5 L min^−1^. The flow rate through the membrane was determined by weighing the permeate on a balance (Kern, Berlin, Germany) connected to a PC. Prior to the experiments, the membranes were compacted at 20–40% higher pressure for 3.5 h, and then the working pressure was adjusted to 10 bar. To determine the separation efficiency and water permeability, all experiments were performed at a temperature of 20.0 °C. The buffer used to maintain pH 7 of the N-nitrosamine solutions was prepared from potassium dihydrogen phosphate (Lach-Ner, Neratovice, Czech Republic, MW: 136.09 g mol^−1^) and dipotassium hydrogen phosphate (VWR Chemicals BDH Prolabo, Rosny-sous-Bois, France, MW: 174.18 g mol^−1^). After membrane compaction and stabilization phase, water flux was measured for at least one hour. Salt rejection was determined by measuring the conductivity of the permeate and feed solutions (Lab 960, SCHOTT Instruments, Mainz, Germany). The initial concentration of the salt solution was 500 mg L^−1^ and 2000 mg L^−1^ for NaCl and CaCl_2_, respectively. To determine the rejection factor for each *N*-nitrosamine, solutions with an initial concentration of 3 mg L^−1^ were prepared in DI, and the concentration was measured by HPLC. To calculate the rejection factor for each marker used for PSD estimation, solutions were prepared with an initial concentration of 100 mg L^−1^, and the concentration before and after membrane filtration was determined using a Total Organic Carbon Analyzer (TOC-VWS, Shimadzu, Kyoto, Japan). The rejection factors (*R*) were calculated using Equation (1), where *c*_p_ and *c*_f_ represent the concentrations of solutes in the permeate and feed solution, respectively.

(1) $$R=1-\frac{c_{p}}{c_{f}}$$

#### 4.3.2. ATR-IR Spectral Analysis

Fourier transform infrared (FTIR) was used to determine the chemical structures of RO membranes. FTIR spectra were recorded at room temperature using a Perkin-Elmer Spectrum One equipped with an ATR module in the frequency range of 650–4000 cm^−1^ with 4 scans and a resolution of 4 cm^−1^. Membrane samples were dried for 24 h prior to analysis. The FTIR spectra were processed using OriginPro 8.5.0 SR1 software.

#### 4.3.3. Contact Angle

Contact angle measurements were performed using the OCAH 200 Data Physics contact angle system. In the sessile drop method, 5 μL of water droplets were applied to the surface of various RO membranes at room temperature. To minimize influences that may affect the contact angle, such as temperature and humidity, the measurements were performed at the same time. The contact angle values obtained for each membrane were the average values of 10 measurements.

#### 4.3.4. Atomic Force Microscopy (AFM)

AFM images of membranes were obtained with the Nanosurf CoreAFM device at room temperature in non-contact acquisition mode (dynamic mode) using a silicon sensor (Tap300 Al-G) with a radius of 10 nm and a vibration frequency of 300 kHz on a surface of 20 × 20, 50 × 50, and 100 × 100 µm, with an acquisition time of 0.78 s. The images were processed using the Gwyddion program. The surface roughness (*R*_q_) is the square root of the sum of the squares of the individual heights and depths from the mean line. Therefore, small variations in height could greatly affect the value of the surface roughness itself. The surface roughness was calculated at about 10 locations in the sample to achieve the highest possible accuracy of the calculated values.

#### 4.3.5. Scanning Electron Microscopy

Dried membrane samples were previously coated with Au/Pd alloy in argon plasma for 90 s to improve their electrical conductivity. Microscopic imaging was performed using a TESCAN Vega3 SEM Easyprobe electron microscope at voltage 10 kV. 

#### 4.3.6. High-Performance Liquid Chromatography Analysis

Quantitative determination of selected *N*-nitrosamines in feed solutions, permeate, and retentate was performed using an Agilent Series high-performance liquid chromatography (HPLC) system coupled with a diode array (DAD) detector (Agilent, Santa Clara, CA, USA). The HPLC system consists of a mobile phase container, a vacuum degasser, an automatic sampler, a thermostated chromatographic column compartment, and a binary pump for mobile phase delivery. Chromatographic separation was performed on a Kinetex C18 chromatographic column (Phenomenex, 150 mm × 4.6 mm, 5 mm, 100 Å) using mobile phase containing MilliQ water as eluent A and acetonitrile as eluent B in gradient elution mode. The gradient elution started with 95% of eluent A, and after 7.5 min of isocratic hold, the proportion of eluent A linearly decreased to 40% over 7.5 min. The proportion of eluent A of 40% was kept unchanged during the next 10 min. In the next 5 min, the proportion of eluent A was returned to the initial value (95%) and stayed the same for 10 min (equilibration). The flow rate of 0.5 mL min^−1^ was maintained throughout the analysis. An injection volume of 20 μL was used in all analyses. Targeted *N*-nitrosamines were detected at the wavelength of 230 nm. Instrument control, data acquisition, and evaluation were performed with ChemStation Rev. B.04.02 SP1 software (Agilent, Santa Clara, USA).

Method limits of quantification (LOQs) estimated experimentally as the lowest concentration with satisfactory precision (RSD < 10%) and trueness (recovery < ±10%) were 10 μg L^−1^ for NDPA and 55 μg L^−1^ for NDEA and NDBA. Calibration curves were linear over the range from LOQ to 10 mg L^−1^ with a coefficient of determination (*R*^2^) higher than 0.999. The obtained LOQ values guarantee the quantification of 0.3% of the initial concentration of NDPA, and 1.8% of the initial concentration of NDEA and NDBA, i.e., a rejection of 99.7% for NDPA and 98.2% for NDEA and NDBA. In cases when nitrosamines were not detected in the samples, a concentration of nitrosamines equal to ½ LOQ was assumed for the calculation of the rejection factors. 

#### 4.3.7. Pore Size Distribution

To calculate the pore size distribution, we used the Surface Force–Pore Flow (SF-PF) theoretical model, first developed by Sourirajan and Matsuura and described elsewhere [50,51,52]. This model assumes that the pores within the PA layer are cylindrical, while the solute–membrane interactions with respect to water can be described as Lennard-Jones surface potential functions representing intermolecular repulsion and dispersion. The calculation procedure searches for a pore size distribution that yields a minimum deviation between the measured and predicted rejections and permeation velocities of selected disc-shaped molecules.

## 5. Conclusions

The selected RO membranes showed pure water permeability in the range of 18–21 L m^−2^ h^−1^ for the UTC73AC and ACM1 membranes, while the highest pure water permeability was determined for the BW30LE membrane (59.4 L m^−2^ h^−1^). The difference in pure water permeability for the pristine membranes is a consequence of the different structure of the PA layer and its different pore sizes and distributions, as shown by PSD. All the selected membranes showed good rejection of NTRs, which was in the range of 75.5–97.4%. However, the rejection factor of NDEA was found to be affected by both solute parameters and pore size distribution. A bimodal pore size distribution was observed for all treated membranes. Treatment of the membrane with *n*-propanol had a positive effect on the water permeability of the UTC73AC membrane (98% increase in water permeability), while the effect on the rejection of NTRs was minimal. However, in the case of ACM1 and BW30LE membranes, treatment with *n*-PrOH decreased water permeability. Moreover, it was shown that the change in the water permeability of the treated membranes was attributed to the rearrangement of polymer molecules, i.e., a change in PSD and a change in the effective number of pores. Treatment of selected membranes with *n*-PrOH resulted in an increase in surface roughness for all membranes. However, the water contact angle increased for the ACM1 and UTC73AC membranes, while it decreased for the BW30LE membranes due to the contribution of other surface parameters.

## Figures and Tables

**Table 1:** Comparison of pore sizes of commercial and *n*-PrOH-treated membranes, radii, and rejection of NDEA, NDPA, and NDBA (“M” stands for membranes after exposure to *n*-PrOH).

Membranes	Pore Size/nm		Rejection/%		
			NDEA (*r* = 0.278 nm)	NDPA (*r* = 0.295 nm)	NDBA (*r* = 0.302 nm)
UTC73AC	0.20–0.63		88.9	94.9	94.9
UTC73AC_M	0.20–0.40	0.47–0.58	85.9	95.8	97.6
ACM1	0.49–0.56		93.8	97.4	95.6
ACM1_M	0.20–0.40	0.49–0.59	93.4	93.2	97.5
BW30LE	0.25–0.40	0.47–0.57	75.5	89.6	86.1
BW30LE_M	0.25–0.40	0.50–0.61	86.4	95.1	98.9

**Table 2:** Membrane roughness (RMS, *S*_q_) for the pristine and *n*-PrOH-treated membranes. (“M” stands for membranes after exposure to *n*-PrOH).

Membrane	UTC73AC	UTC73AC_M	ACM1	ACM1_M	BW30LE	BW30LE_M
RMS/nm	37.3	48.0	53.3	86.8	52.8	58.4

**Table 3:** Nominal membrane characteristics (manufacturer’s data).

	DOW-FILMTEC™ BW30LE	Toray™ UTC-73AC	TriSep™ ACM1
Feed	Brackish Water	Brackish Water	Brackish Water
Type	Low Energy	High Rejection, Low Energy, Cl Resistant	“Tight”
pH Range (25 °C)	2–11	2–11	2–11
Flux ^1^/L m^−2^ h^−1^ bar^−1^	4.09–5.09	3.40	2.77
Rejection (NaCl)	99.0%	99.8%	99.5%
Pore size/MWCO	N/A	N/A	N/A
Polymer	Polyamide-TFC	Polyamide-TFC	Polyamide-TFC

^1^ The performance of all membranes was tested at an operating pressure of 15.5 bar.

## Data Availability

Data sharing is not applicable to this article.
